# S1P, dihydro-S1P and C24:1-ceramide levels in the HDL-containing fraction of serum inversely correlate with occurrence of ischemic heart disease

**DOI:** 10.1186/1476-511X-10-70

**Published:** 2011-05-09

**Authors:** Kelley M Argraves, Amar A Sethi, Patrick J Gazzolo, Brent A Wilkerson, Alan T Remaley, Anne Tybjaerg-Hansen, Børge G Nordestgaard, Sharon D Yeatts, Katherine S Nicholas, Jeremy L Barth, W Scott Argraves

**Affiliations:** 1 Department of Regenerative Medicine and Cell Biology, Medical University of South Carolina, Charleston, SC 29425 USA; 2 Lipoprotein Metabolism Section, Vascular Medicine Branch, National Heart, Lung, and Blood Institute, National Institutes of Health, Bethesda, MD 20892 USA; 3 Department of Clinical Biochemistry, Rigshospitalet, Copenhagen University Hospital, Faculty of Health Sciences, University of Copenhagen, Denmark; 4 Department of Clinical Biochemistry, Herlev Hospital, Copenhagen University Hospital, Faculty of Health Sciences, University of Copenhagen, Denmark; 5 The Copenhagen City Heart Study, Bispebjerg Hospital, Copenhagen University Hospital, Faculty of Health Sciences, University of Copenhagen, Denmark; 6 Division of Biostatistics and Epidemiology, Medical University of South Carolina, Charleston, SC 29425 USA

## Abstract

**Background:**

The lysosphingolipid sphingosine 1-phosphate (S1P) is carried in the blood in association with lipoproteins, predominantly high density lipoproteins (HDL). Emerging evidence suggests that many of the effects of HDL on cardiovascular function may be attributable to its S1P cargo.

**Methods:**

Here we have evaluated how levels of S1P and related sphingolipids in an HDL-containing fraction of human serum correlate with occurrence of ischemic heart disease (IHD). To accomplish this we used liquid chromatography-mass spectrometry to measure S1P levels in the HDL-containing fraction of serum (depleted of LDL and VLDL) from 204 subjects in the Copenhagen City Heart Study (CCHS). The study group consisted of individuals having high serum HDL cholesterol (HDL-C) (females:≥73.5 mg/dL; males:≥61.9 mg/dL) and verified IHD; subjects with high HDL-C and no IHD; individuals with low HDL-C (females:≤38.7 mg/dL; males:≤34.1 mg/dL) and IHD, and subjects with low HDL-C and no IHD.

**Results:**

The results show a highly significant inverse relationship between the level of S1P in the HDL-containing fraction of serum and the occurrence of IHD. Furthermore, an inverse relationship with IHD was also observed for two other sphingolipids, dihydro-S1P and C24:1-ceramide, in the HDL-containing fraction of serum. Additionally, we demonstrated that the amount of S1P on HDL correlates with the magnitude of HDL-induced endothelial cell barrier signaling.

**Conclusions:**

These findings indicate that compositional differences of sphingolipids in the HDL-containing fraction of human serum are related to the occurrence of IHD, and may contribute to the putative protective role of HDL in IHD.

## Background

The lysosphingolipid, sphingosine 1-phosphate (S1P), is a component of human plasma [1]. Approximately 65% of the S1P in blood is associated with the lipoproteins LDL, VLDL and HDL, with the majority of lipoprotein-associated S1P (~85%) bound to HDL [2]. Findings from a growing number of animal and *in vitro *studies suggest that S1P is a mediator of many of the cardiovascular effects of HDL, including its ability to promote vasodilation, angiogenesis and endothelial barrier function, to protect against ischemia/reperfusion injury and to inhibit/reverse atherosclerosis [3,4]. These latter, potentially cardioprotective, effects involve S1P-mediated suppression of various inflammatory processes, including the reduction of endothelial expression of monocyte and lymphocyte adhesion molecules and decreased recruitment of polymorphonuclear cells to sites of myocardial infarction [3,4]. The S1P-dependent enhancement of endothelial barrier activity by HDL [5] may also be cardioprotective considering that reduction in endothelial barrier is a factor underlying post ischemic edema, the recruitment and migration of monocytes as well as the introduction of triglyceride rich lipoprotein particles into the blood vessel intima [6].

Epidemiological data from the Framingham Heart Study [7,8] and other prospective studies [9] demonstrate that high levels of HDL cholesterol (HDL-C) in blood are inversely associated with risk for cardiovascular disease. However, some individuals with high HDL-C and normal LDL cholesterol (LDL-C) still develop cardiovascular disease [10]. This has lead to the hypothesis that the HDL in some individuals might be dysfunctional as an anti-atherogenic agent or perhaps even proatherogenic as a result of the HDL lipid content, particularly that of S1P. In the present study, we addressed this hypothesis by measuring levels of S1P and related sphingolipids in HDL-containing fractions of serum from groups of individuals having either high or low HDL-C, with or without occurrence of ischemic heart disease (IHD).

## Results

### Inverse correlation of S1P, DH-S1P and C24:1-ceramide in the HDL-containing fraction of serum with ischemic heart disease

Subjects were categorized into four groups based on having high or low HDL-C and the presence or absence of IHD (Table 1). Serum from each subject was subjected to dextran sulfate/MgCl_2 _precipitation to prepare a serum fraction containing HDL but depleted of LDL and other apoB-containing lipoproteins. Results of liquid chromatography/mass spectrometry (LC-MS-MS) composition analysis of sphingolipids in HDL-containing fractions are summarized in Table 2. The major sphingolipids associated with the HDL-containing fractions were S1P, C24 ceramide and C24:1 ceramide. As compared to total serum (Table 3), the HDL-containing fraction contained ~76% of the S1P, 21% of the C24 ceramide and 20% of the C24:1 ceramide found in total serum.

**Table 1:** Characteristics of participants with and without IHD from the Copenhagen University Hospital and The Copenhagen City Heart Study.

	**With IHD**		**No IHD**	
	
	**High HDL-C** **(*n*= 53)** **Group 1**	**Low HDL-C** **(*n*= 42)** **Group 2**	**High HDL-C** **(*n*= 55)** **Group 3**	**Low HDL-C** **(*n*= 54)** **Group 4**
	
Age, years	63.1 ± 10.3	61.5 ± 9.3	62.6 ± 10.3	62.7 ± 9.6
Total cholesterol, mg/dL	208.1 ± 25.7	182.3 ± 29.4	207.3 ± 31.2	166.8 ± 31.1
High density lipoprotein-C, mg/dL	78.4 ± 14.3	32.4 ± 5.2	80.5 ± 14.1	33.6 ± 5.7
Low density lipoprotein-C, mg/dL	113.4 ± 26.3	120.9 ± 30.6	118.8 ± 28.0	117.7 ± 25.6
Triglycerides, mg/dL	82.1 ± 30.0	104.9 ± 31.1	74.1 ± 25.2	107.8 ± 29.4
Body mass index, kg/m^2^	24.8 ± 4.2	26.0 ± 3.5	23.6 ± 3.1	28.0 ± 5.0
Smokers, %	27.1	26.8	42.6	33.3
Diabetes mellitus, %	7.7	14.3	5.5	7.4

All values are original measurements from the above-mentioned studies. Selection and matching for the present study were based on these values. All individuals had LDL-C <160 mg/dL, triglycerides <150 mg/dL, and none were treated with LDL-C-lowering medications. Group 1: Females n = 16 Males n = 37; Group 2: Females n = 13 Males n = 29; Group 3: Females n = 16 Males n = 39; Group 4: Females n = 15 Males n = 39. Group 1 had high HDL-C (>90th percentile) and verified IHD; this group was compared with Group 3 without IHD, but matched by age, sex, and similar HDL-C levels (>90th percentile HDL-C levels for Group 1 and 3 individuals were females:≥ 73.5 mg/dL; males:≥ 61.9 mg/dL). Group 2 had low HDL-C (<10th percentile) and verified IHD; this group was compared with Group 4 without IHD, but matched by age, sex, and similar HDL-C levels (<10th percentile HDL-C levels for Group 2 and 4 individuals were females:≤ 38.7 mg/dL; males:≤ 34.1 mg/dL). All individuals without IHD were selected from The Copenhagen City Heart Study's 4th examination. Patients with IHD were selected from individuals referred to the Copenhagen University Hospital, Rigshospitalet, Denmark for coronary angiography.

**Table 2:** LC-MS-MS analysis of sphingolipid composition in HDL-containing fractions from CCHS serum samples.

**Sphingolipid**^1^	Concentration (nM)									
	
	**High HDL-C, IHD**^**2**^		**High HDL-C, No IHD**^**2**^			**Low HDL-C, IHD**^**2**^		**Low HDL-C, No IHD**^**2**^		
	Mean	Std	Mean	Std	**P value**^3^	Mean	Std	Mean	Std	**P value**^3^
S1P	1362.7	349.6	1643.4	256.2	<0.0001	1220.0	279.6	1569.1	357.0	<0.0001
Sph	10.4	8.5	BDL		NA	BDL		BDL		NA
DHSph	BDL		BDL		NA	BDL		9.9	22.9	NA
DH-S1P	95.4	49.1	150.5	42.1	<0.0001	82.1	38.1	122.3	45.5	<0.0001
C16-Cer	45.2	60.2	25.6	10.1	<0.0001	BDL		19.7	28.9	NA
DHC16-Cer	9.4	11.5	6.9	3.5	0.5122	BDL		BDL		NA
C14-Cer	BDL		BDL		NA	BDL		BDL		NA
C18:1-Cer	15.7	5.2	BDL		NA	BDL		BDL		NA
C18-Cer	42.2	14.4	41.8	15.9	0.9872	36.9	8.1	34.2	6.6	0.4587
C20:1-Cer	26.9	11.8	BDL		NA	BDL		BDL		NA
C20-Cer	103.6	52.3	94.9	37.4	0.8361	43.1	19.7	43.8	17.7	0.9542
C22-Cer	168.5	94.3	208.1	95.8	0.0999	65.4	39.6	79.9	47.7	0.2406
C20:4-Cer	10.1	4.0	BDL		NA	BDL		BDL		NA
C22:1-Cer	61.0	32.3	60.7	33.9	0.9851	19.2	8.0	24.9	9.1	0.0223
C24-Cer	1253.8	843.0	2008.3	858.6	<0.0001	708.2	309.9	785.8	392.2	0.8214
C24:1-Cer	699.1	437.5	912.4	348.8	0.0002	320.6	152.5	435.4	163.1	0.0027
C26-Cer	47.1	186.4	38.5	81.0	<0.0001	7.6	3.9	BDL		NA
C26:1-Cer	36.5	106.3	27.1	16.2	0.0008	8.4	4.6	8.4	5.4	0.9629

^1^Abbrevations used: Sph, sphingosine; DH, dihydro; Cer, ceramide; P, phosphate; BDL, below detection limit, NA, not applicable since at least one group in the pair was below detection limit.

^2^Sample sizes are High HDL-C, IHD, n = 53; High HDL-C, no IHD, n = 55; Low HDL-C, IHD, n = 42; Low HDL-C, no IHD, n = 54.

^3^p-values are derived from a Tukey's comparison following a one-way ANOVA test conducted at level of significance 0.05.

**Table 3:** LC-MS-MS analysis of sphingolipid composition in total serum fractions from CCHS serum samples.

**Sphingolipid**^**1**^	Concentration (nM)									
	
	**High HDL-C, IHD**^**2**^		**High HDL-C, No IHD**^**2**^			**Low HDL-C, IHD**^**2**^		**Low HDL-C, No IHD**^**2**^		
	Mean	Std	Mean	Std	**P value**^3^	Mean	Std	Mean	Std	**P value**^3^
S1P	1889.9	546.0	1849.8	407.6	O-NS	1879.2	549.0	2053.0	628.0	O-NS
Sph	BDL		22.3	11.0	NA	16.0	11.6	52.3	59.9	<0.0001
DHSph	BDL		14.8	9.2	NA	10.1	5.1	16.6	11.6	<0.0001
DH-S1P	213.0	76.1	276.8	96.5	0.0032	273.9	98.2	271.3	106.0	0.9992
C16-Cer	171.4	70.7	212.0	86.8	0.0276	360.1	163.9	222.5	78.7	<0.0001
DHC16-Cer	139.1	74.9	60.1	38.5	<0.0001	77.1	39.7	75.5	31.6	0.9958
C14-Cer	54.6	8.9	92.3	19.7	<0.0001	117.0	34.4	103.0	35.1	0.0412
C-18:1-Cer	91.6	33.3	49.5	13.5	<0.0001	63.7	25.6	65.2	13.6	0.5492
C18-Cer	163.8	51.0	123.9	42.3	0.0002	171.2	69.9	123.2	31.9	<0.0001
C20:1-Cer	57.5	38.5	17.4	6.1	<0.0001	28.4	15.4	25.0	9.7	0.9997
C20-Cer	481.1	205.9	249.9	94.2	<0.0001	320.9	153.3	270.8	120.0	0.3547
C22-Cer	1045.2	347.4	758.2	194.0	<0.0001	1116.0	420.3	915.4	317.9	0.0147
C20:4-Cer	BDL		BDL		NA	BDL		6.7	10.1	NA
C22:1-Cer	128.7	118.9	62.3	37.3	0.0061	206.5	160.1	61.4	47.9	<0.0001
C24-Cer	6026.3	1816.2	5522.0	1381.9	O-NS	5577.5	2080.1	5724.1	1715.1	O-NS
C24:1-Cer	3433.4	847.9	2424.3	545.8	<0.0001	2838.6	951.2	3223.2	688.7	0.0681
C26-Cer	40.7	32.8	33.8	15.7	O-NS	38.9	30.2	36.0	18.8	O-NS
C26:1-Cer	30.7	20.0	30.8	11.9	0.8093	40.3	21.2	29.8	15.9	0.0140

^1^Abbrevations used: Sph, sphingosine; DH, dihydro; Cer, ceramide; P, phosphate; BDL, below detection limit; O-NS, Overall not significant by ANOVA; NA, not applicable since at least one group in the pair was below detection limit.

^2^Sample sizes are High HDL-C, IHD, n = 53; High HDL-C, no IHD, n = 55; Low HDL-C, IHD, n = 42; Low HDL-C, no IHD, n = 54.

^3^p-values are derived from a Tukey's comparison following a one-way ANOVA test conducted at level of significance 0.05.

**Figure 1:**
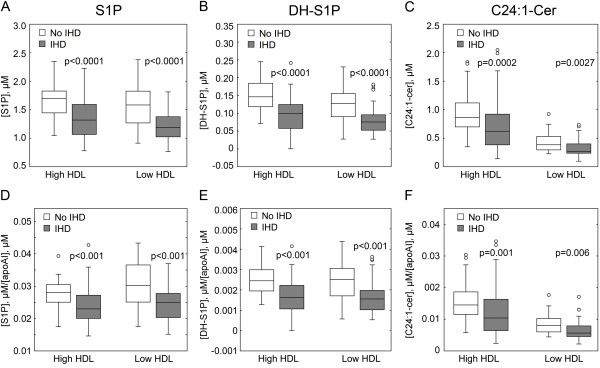
**An inverse correlation exists between the occurrence of IHD and levels of S1P and DH-S1P in HDL-containing fractions from CCHS subject serum**. S1P (***A***) DH-S1P (***B***) and C24.1 ceramide (***C***) levels were measured by blinded LC-MS-MS analysis of 55 HDL-containing preparations from CCHS individuals with high HDL-C and no evidence of IHD, 53 samples from individuals with high HDL-C and evidence of IHD, 54 samples from individuals with low HDL-C and no evidence of IHD and 42 samples from individuals with low HDL-C and evidence of IHD. In ***D-F***, levels of S1P and DH-S1P in HDL-containing serum fractions from CCHS individuals with and without IHD are assessed relative to apoA-I content. Levels of apoA-I in samples were quantified by immunoturbidometric assay using a Cobas Fara analyzer. Indicated p-values are derived from a Tukey's comparison following a one-way ANOVA test conducted at level of significance 0.05. Data are presented in box-and-whisker diagrams. For the box-and-whisker diagrams, the boxes correspond to the interquartile range (IQR). The horizontal bar within the box is drawn at the height of the median. The whiskers indicate the range of the data within 1.5 X IQR with outliers indicated as circles.

Statistical analysis of the LC-MS-MS data showed that S1P and DH-S1P in HDL-containing serum fractions were significantly lower (17% and 37% reduction, respectively; p < 0.0001) in individuals with high HDL-C having IHD as compared to individuals with high HDL-C having no evidence of IHD (Figure 1A and 1B). Furthermore, S1P and DH-S1P in HDL-containing fractions were significantly lower (22% and 33% reduction, respectively; p < 0.0001) in individuals with low HDL-C and having IHD as compared to individuals with low HDL-C having no evidence of IHD (Figure 1A and 1B).

**Figure 2:**
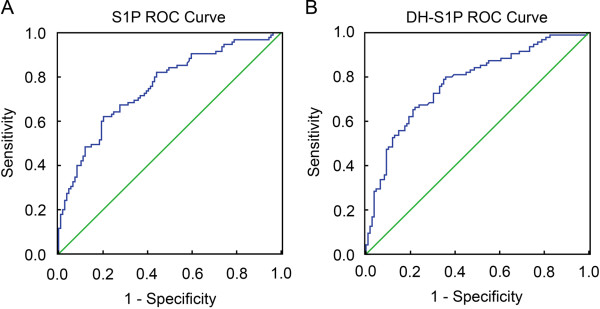
**Receiver operating characteristic (ROC) curves for the ability of S1P and DH-S1P to distinguish between subjects with and without IHD**. Smaller values of the corresponding measure indicate stronger evidence for the presence of IHD. ROC curves were constructed using SPSS v16.

The ability to correctly classify patients with and without IHD by using levels of S1P and DH-S1P was assessed by receiver operating characteristic (ROC) analysis (Figure 2). S1P and DH-S1P levels in the HDL-containing fraction were both found to discriminate subjects with IHD from those without IHD better than would be expected by chance alone, as characterized by the area under the curves (0.756 for S1P and 0.773 for DH-S1P) (Figure 2).

Among the other sphingolipids analyzed in the HDL-containing fractions from the CCHS subjects, C24:1-ceramide levels were also significantly lower (p < 0.003) in individuals with IHD as compared to their control groups for both the high and low HDL groups (Figure 1C).

S1P, DH-S1P and C24:1-ceramide levels in the HDL-containing fractions were also assessed relative to the concentration of apolipoprotein A-I (apoA-I) in the samples. The ratios of these sphingolipids to apoA-I were all significantly lower (p ≤ 0.001) in individuals with high HDL-C having IHD as compared to individuals with high HDL-C having no evidence of IHD (Figure 1D-F). Furthermore, these ratios were also significantly lower (p ≤ 0.006) in samples from individuals with low HDL-C and having IHD as compared to individuals with low HDL-C having no evidence of IHD (Figure 1D-F).

### The proportion of subjects with IHD decreases as [S1P] and [DH-S1P] increase

To examine the quantitative relationship of the concentration of these lysosphingolipids associated with the HDL-containing fraction and IHD, subjects were grouped as shown in Table 4 by their quartile of [S1P] in the HDL-containing fraction of serum (upper limits for Q1 = 1.21, Q2 = 1.45, Q3 = 1.73, Q4 = 2.38 μM). A Cochran-Armitage test for linear trend was conducted to determine whether an increase in [S1P] corresponded to a decrease in the proportion of subjects with IHD. The result was statistically significant (Z = 6.0887, p-value < 0.0001), indicating that the proportion of subjects with IHD decreases as [S1P] (as defined by the corresponding quartile) increases. The analysis was repeated using quartiles of [DH-S1P] (upper limits for Q1 = 0.075, Q2 = 0.113, Q3 = 0.149, Q4 = 0.244 μM) (Table 5). The result of the Cochran-Armitage test of these data was also highly statistically significant (Z = 6.4654, p-value < 0.0001), indicating that the proportion of subjects with IHD decreases as [DH-S1P] (as defined by the corresponding quartile) increases.

**Table 4:** Contingency table of [S1P] quartiles and IHD status.

Quartile	[S1P], μM	Percentage of Subjects (n)		Number of Subjects
			
		No IHD	IHD	
1	0.764 < [S1P] ≤ 1.206	21.6 (11)	78.4 (40)	51
2	1.206 < [S1P] ≤ 1.451	51.0 (26)	49.0 (25)	51
3	1.451 < [S1P] ≤ 1.731	58.8 (30)	41.2 (21)	51
4	1.731 < [S1P] ≤ 2.376	82.4 (42)	17.7 (9)	51

Totals		109	95	204

**Table 5:** Contingency table of [DH-S1P] quartiles and IHD status.

Quartile	[DH-S1P], μM	Percentage of Subjects (n)		Number of Subjects
			
		No IHD	IHD	
1	0.000 < [DH-S1P] ≤ 0.075	19.6 (10)	80.4 (41)	51
2	0. 075 < [DH-S1P] ≤ 0.113	45.1 (23)	54.9 (28)	51
3	0. 113 < [DH-S1P] ≤ 0.149	70.6 (36)	29.4 (15)	51
4	0.149 < [DH-S1P] ≤ 0. 244	78.4 (40)	21.6 (11)	51

Totals		109	95	204

### Analysis of sphingolipids in total serum samples

Sphingolipids in total serum from the CCHS subjects were also evaluated. As shown in Table 3, levels of S1P in total serum were not significantly different between IHD and non IHD individuals for either the high HDL-C or low HDL-C groups. Among the other sphingolipids analyzed in total serum, C18-Cer, C22-Cer and C22:1-Cer were significantly higher in the total serum from individuals with IHD as compared to individuals without IHD irrespective of their HDL levels (Table 3). Levels of several other sphingolipids were statistically different between IHD and non IHD individuals in the high HDL-C and low HDL-C groups, but the direction of the difference was opposite between the two groups.

### DH-S1P enhances endothelial barrier activity in an S1P1-dependent manner

**Figure 3:**
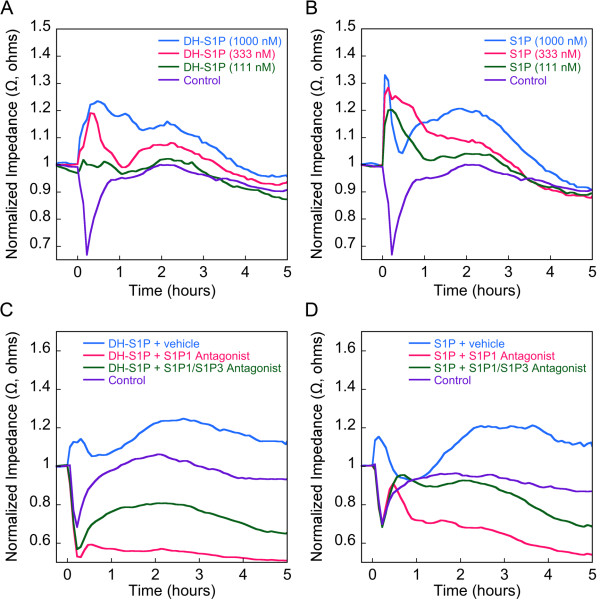
**DH-S1P enhances endothelial barrier in an S1P1 dependent manner**. Confluent EC monolayers were grown under serum-free conditions until a minimal TEER plateau was reached. In ***A***, EC monolayers were incubated with varying concentrations of DH-S1P [111-1000 nM]. In ***B***, EC monolayers were incubated with varying concentrations of S1P [111-1000 nM]. In ***C***, EC monolayers were incubated with DH-S1P [1000 nM] plus and minus the S1P1 antagonist W146 or the S1P1/S1P3 antagonist VPC23019 (each at 10 μM). In ***D***, EC monolayers were incubated with S1P [1000 nM] plus and minus the S1P1 antagonist W146 or the S1P1/S1P3 antagonist VPC23019 (each at 10 μM). Each of the TEER tracings shown is an average from three independent experiments each with two replicates per condition. Impedance values were normalized by dividing each value by the level of impedance measured just prior to the addition of effectors. As a control for *A *and *B*, monolayers were treated with 40 μg/ml delipidated albumin (Control), a concentration corresponding to the amount of BSA carrier used for the highest concentration of DH-S1P and S1P tested. As controls for *C *and *D*, ECs were treated with delipidated albumin-containing serum free medium (SFM) plus vehicle buffer.

While the effects of S1P on endothelial cell functions have been implicated as a basis for the anti-atherogenic effects of HDL, rather little is known as to the effects of DH-S1P on vascular cell behaviors. Generally, DH-S1P has been found to be an agonist of S1P receptors [11-13]. We used electrical cell substrate impedance sensing (ECIS) to assess the ability of DH-S1P to influence endothelial barrier activity, a major physiological function of the endothelium. DH-S1P (on an albumin carrier) induced a dose-dependent increase in transendothelial electrical resistance (TEER) (Figure 3A). The magnitude of the response was similar to that achieved using S1P (Figure 3B). By contrast, C24:1-ceramide, the third sphingolipid found to be significantly lower in HDL-containing fractions of serum from individuals with IHD, did not effect TEER (Additional file 1, ***Figure S1***).

**Figure 4:**
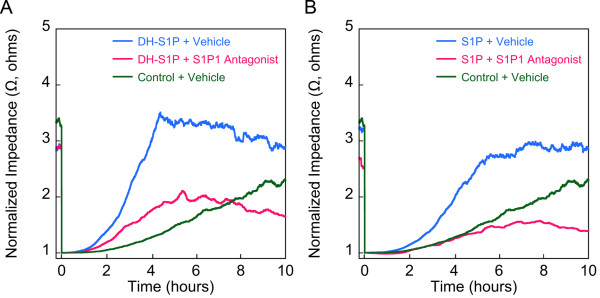
**DH-S1P enhances endothelial cell motility in an S1P1 dependent manner**. EC monolayers were wounded with a burst of high electrical current as described previously [5] and the culture medium then supplemented with DH-S1P (1 μM) (***A***), or S1P (1 μM) (***B***) in the presence or absence of the S1P1 antagonist W146 (10 μM in DMSO vehicle). The migration of cells into the wounded areas was measured in real-time by electrical impedance. As a control in both experiments the medium was supplemented with delipidated BSA (Control) in PBS. Electrical impedance data are normalized to baseline following wounding. The data depicted are representative of two independent experiments and traces represent averages of two replicates per condition.

We next evaluated the effects of S1P1 receptor antagonists on the process of DH-S1P-induced barrier enhancement. The S1P1 antagonist, W146, and the S1P1/S1P3 antagonist, VPC23019, both inhibited the TEER response to DH-S1P, similar to the inhibitory effects these agents exert on S1P-mediated barrier enhancement (Figure 3C and 3D). ECIS analyses were also used to evaluate the effect of DH-S1P on endothelial cell migration. The results showed that DH-S1P also promoted S1P1-dependent endothelial cell migration (Figure 4).

### Enrichment of HDL with S1P elicits a dose dependent enhancement of endothelial barrier function

**Figure 5:**
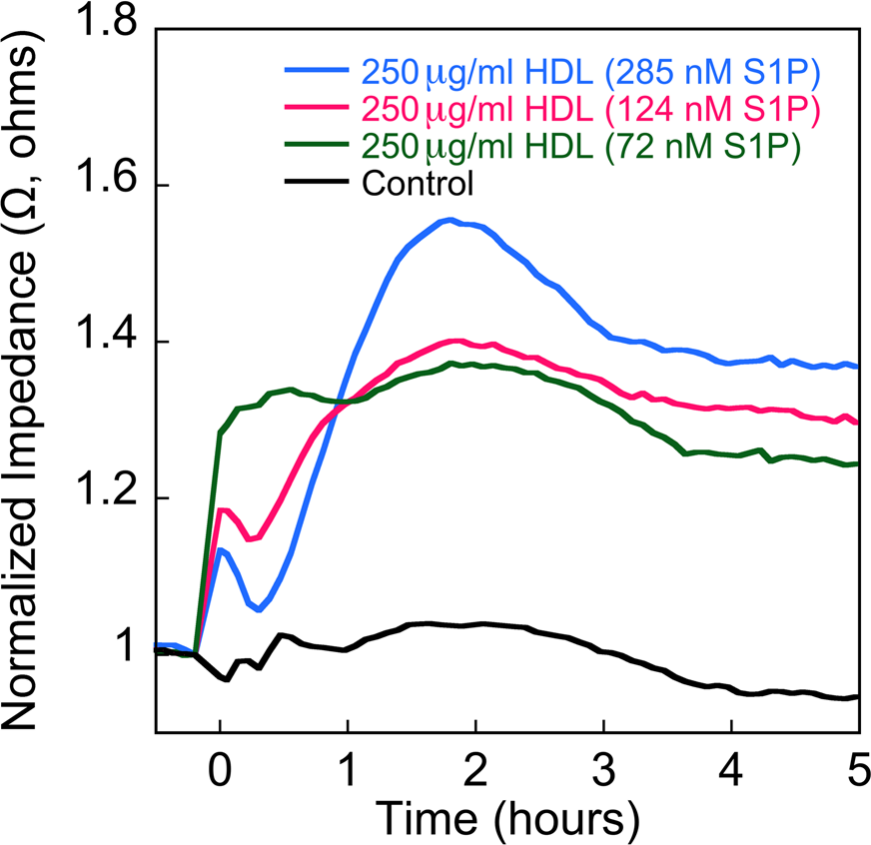
**Levels of HDL-associated S1P dictate the magnitude of the TEER response**. Confluent EC monolayers were incubated with native HDL (72 nM S1P) or HDL containing varying amounts of exogenously added S1P. HDL was added to achieve a final concentration of 250 μg protein/ml. As a control, monolayers were incubated with the vehicle buffer, 0.03 mM EDTA in Dulbecco's PBS (Control). Each of the TEER tracings shown is an average from two replicates per treatment. Impedance values were normalized by dividing each value by the level of impedance measured just prior to the addition of effectors. The results depicted are representative of two independent experiments.

Findings from the LC-MS-MS analysis of samples from subjects with and without IHD suggested that S1P and DH-S1P levels on HDL particles might relate to the biological activity of HDL in the context of its anti-atherogenic effects. To address this we evaluated the effect of HDL enriched with varying amounts of S1P on endothelial cell barrier function. Transendothelial electrical resistance monitoring showed that HDL enriched with S1P elicited a dose dependent increase in maximal impedance and sustained a higher level of impedance than native HDL (Figure 5). These results are consistent with the possibility that subjects with higher levels of HDL-associated S1P might have lower vascular permeability, which would be expected to decrease their susceptibility to atherosclerosis.

## Discussion

This study was undertaken to test whether differences in sphingolipid content of HDL might relate to the putative protective role of HDL in IHD. The findings indicate that levels of S1P, DH-S1P and C24:1-ceramide in the HDL-containing fraction of serum are inversely related to the occurrence of IHD. This inverse relationship was found to exist regardless of whether the patients had high or low HDL-C levels. As an evidence of the potential functional significance of these findings we demonstrated that endothelial barrier response is sensitive to the levels of HDL-associated S1P. Alteration in endothelial barrier function is a critical factor underlying post ischemic edema, recruitment and migration of monocytes, and introduction of triglyceride rich lipoprotein particles into the intima of blood vessels [6,14]. Thus, our findings support the hypothesis that the atheroprotective activity of HDL is at least in part a function of S1P content, with higher levels being protective.

Our findings raise a number of questions that require further investigation. First, our studies do not precisely define the blood carrier of S1P or DH-S1P important for the IHD correlation that we have observed. While the dextran sulfate/MgCl_2 _precipitation procedure that we employed effectively removes LDL and other apoB-containing lipoproteins, but not HDL from the serum, it also does not remove albumin, a known blood carrier of S1P [2]. Epidemiological studies have shown that a highly significant inverse relationship exists between serum albumin levels and risk of coronary heart disease [15]. Furthermore, most of the putative cardioprotective effects reported for S1P have been experimentally demonstrated using albumin as the S1P carrier [3,5]. Thus, there is a future need to establish whether HDL-S1P and/or albumin-S1P levels in blood correlate inversely with occurrence of IHD. In this regard, it is important to point out that the effect of HDL-associated S1P on barrier function has been shown to persist longer than an equimolar dose of albumin-associated S1P [5] as well as the fact that HDL carries the majority of blood borne S1P (~55%) [2].

The observed values of S1P in serum and HDL-containing fractions of CCHS samples (from subjects with and without IHD) are generally higher than serum values reported in the literature, which range from 484 ± 82 pmol/ml [1] to 1035 ± 26.4 pmol/ml [16]. It is possible that erythrocyte hemolysis might have contributed to the elevated level of S1P, since red cells contain stores of S1P [17,18]. However, there was no overt evidence that red cell hemolysis had occurred during the process of blood collection and serum preparation. Despite the fact that the measured S1P values were generally high in the CCHS samples, the interpretations made in the present study are in agreement with findings from a recent report by Sattler et al. [19] showing that the amount of S1P in isolated HDL was lower in subjects with stable coronary artery disease as compared to controls.

While our analysis showed that an inverse correlation existed between the occurrence of IHD and S1P levels in the HDL-containing fraction (LDL and VLDL depleted fraction), no significant correlation was observed between S1P levels in total serum and the occurrence of IHD. From these observations it follows then that decreases in HDL-associated S1P in IHD subjects are balanced by increases in S1P levels elsewhere, perhaps in LDL and/or VLDL. If so it would be expected that S1P levels in the LDL- and VLDL-containing fraction of serum would correlate directly with IHD in our study population. Despite the fact that total serum S1P levels were not statistically different between IHD and non-IHD subjects in our study we observed that S1P was differentially partitioned in these groups. In non IHD subjects, the HDL containing fraction had 82% of the S1P detected in total serum whereas IHD subjects had only 69% of the S1P detected in total serum (this data can be extracted from Tables 2 and 3). This suggests that the mechanism controlling partitioning of S1P to HDL versus other lipoproteins is acting differently between IHD and non IHD subjects.

Although our study did not detect a correlation between S1P levels in total serum and IHD, another recent study showed a positive correlation between serum S1P levels and coronary artery disease [16]. The apparent disparity may relate to differences in the study populations. In the study by Deutschman et al. [16], 61% of the subjects had elevated LDL-C and "the average patient was taking three prescription drugs for heart or circulatory problems, including β-blockers, lipid-lowering agents, calcium blockers, nitrates, and ACE inhibitors". There is no indication of how these drugs or concentration of LDL-C might influence S1P levels. Indeed, these drugs may augment S1P as part of their therapeutic benefit. Our subject population was not receiving lipid-lowering drugs and had normal LDL-C levels.

While there is much known about the bioactivities of S1P, particularly in the context of vascular biology, relatively little is known as to the biological actions of DH-S1P and C24:1-ceramide, the other two sphingolipids whose levels in the HDL-containing fraction inversely correlate with the occurrence of IHD. Positive correlations have been found between total plasma ceramide levels and levels of total cholesterol and triglycerides [20], well-known lipid risk factors of atherosclerosis [21-23]. While this may seem in opposition to our findings, it is notable that *1) *C24:1-ceramide is a specific subspecies of ceramide, *2) *our study population only included individuals displaying normal levels of total cholesterol and triglycerides (Table 1), and 3) our measurements were made on the HDL-containing fraction of serum, not total plasma or serum. It is not clear how C24:1-ceramide associated with HDL might influence the etiology of IHD. There is no known cell surface receptor for C24:1-ceramide; however, ceramide has been shown to be transported through the action of the ATP-binding cassette (ABC) transporter, ABCA7 [24], which effluxes lipids to apoA-I [25]. This could account for the presence of C24:1-ceramide on HDL, but how or whether this C24:1-ceramide influences the bioactivities of HDL remains to be determined. With respect to DH-S1P, again there is limited information pertaining to its role in influencing vascular cell processes. Here we show that DH-S1P, like S1P, is a potent inducer of S1P1-dependent endothelia cell barrier function and endothelial cell migration. These findings are consistent with reports that DH-S1P acts as an S1P receptor agonist [11-13]. Other studies have shown that DH-S1P can mediate induction of matrix metalloproteinase 1 (MMP1) expression in dermal fibroblasts, a response not reproduced by S1P [26]. MMP1 is believed to play an important role in the pathogenesis of atherosclerosis. Findings from mouse studies indicate that MMP1 can inhibit atherosclerosis [27], and recent human studies show that persons homozygous for a transcriptionally overactive allele of the MMP1 gene have a reduced risk of coronary heart disease [28]. Thus, HDL with low levels of DH-S1P might be less atheroprotective owing to its reduced capacity to induce MMP1 expression.

Another potential implication from this study is that the measurement of S1P and/or related sphingolipids may have diagnostic value. Based on ROC analysis (Figure 2), the sensitivity and specificity of S1P and DH-S1P is similar to what has been previously described for the use of routine lipid and lipoprotein markers for cardiovascular risk assessment [29]. In the present study, the subjects were matched for HDL-C levels, but S1P and DH-S1P still differed between subjects with and without IHD. While additional risk analyses on different cohorts will need to be performed, the results from the present study suggest that the S1P and DH-S1P content of the HDL containing fraction may be helpful in identifying individuals that are at increased risk of IHD regardless of their HDL-C levels.

## Conclusions

Our findings highlight the possibility that the effects of lipoproteins on the etiology of cardiovascular disease may be attributable, at least in part, to sphingolipid composition of lipoproteins. At least one predominantly HDL-associated sphingolipid, S1P, is well known to elicit an array of vascular responses, many of which can be considered as cardioprotective. Furthermore, many of the cardioprotective effects of HDL have been attributed to its S1P cargo. Now through analysis of large numbers of serum samples from human subjects, we have established that levels of S1P and two other sphinoglipids in the HDL-containing fraction of serum have highly significant inverse correlations with the occurrence of IHD. This is evidence that in addition to cholesterol, sphingolipids may be risk factors for IHD as well as targets for therapeutic intervention. If it is established that low levels of HDL-associated sphingolipids are IHD risk factors, then therapies that increase specific plasma HDL-sphingolipid levels may hold promise for decreasing the risk for IHD.

## Methods

### Study group

The study involved the analysis of blood serum samples existing in the Copenhagen City Heart Study (CCHS) collection [21,30] which includes a group of patients with documented coronary atherosclerosis and IHD (the Copenhagen Ischemic Heart Disease Study) [31,32]. The CCHS is a prospective cardiopulmonary study of 20- to 93-year-old Danes of both sexes sampled from the general population in 1976-1978 and reexamined in 1981-1983, 1991-1994 and 2001-2003 [33]. Informed consent was obtained from all participants. The Danish Ethics Committees of Copenhagen and Frederiksberg approved the study (study No. 100.2039/91 and KA93125). Based on analysis of the total population (individuals from CCHS without ischemic heart disease (IHD), n = 5,911 [women = 3,384; men = 2,527]) the average normal level of HDL-C for women is 62.1 ± 18.9 mg/dL and 50.7 ± 16.4 mg/dL for men. Testing was performed on four groups of CCHS samples, including 55 samples from individuals with high HDL-C (80.3 ± 14.3 mg/dL) and no evidence of IHD, 53 samples from gender and age matched individuals with high HDL-C (78.8 ± 14.2 mg/dL) and verified IHD, 54 samples from individuals with low HDL-C (33.7 ± 5.8 mg/dL) and no evidence of IHD, and 42 samples from gender and age matched individuals with low HDL-C (31.8 ± 5.3 mg/dL) and verified IHD. All individuals had LDL-C <160 mg/dL, triglycerides <150 mg/dL, and none were treated with LDL-C-lowering medications. Subjects had an average age of 62 years, approximately 30% were women and 8.5% had diabetes mellitus. The characteristics of subjects from which samples were derived are summarized in Table 1. Additional apolipoprotein and lipid information pertaining to the same groups of subjects as analyzed herein is published in a recent study [34]. This study also showed that for all of the criteria described in Table 1, there was no statistically significant difference between individuals with and without IHD for both the high and low HDL-C groups.

### Sphingolipid analysis of CCHS serum samples

Blinded liquid chromatography-tandem mass spectrometry (LC-MS-MS) sphingolipid analysis was performed on aliquots of total serum samples and on aliquots of LDL- and VLDL-depleted, HDL-containing preparations from CCHS serum samples. To prepare LDL- and VLDL-depleted, HDL-containing preparations, CCHS serum samples were subjected to magnetic bead-dextran-sulfate/MgCl_2 _precipitation (Reference Diagnostics, Inc., Bedford, MA) to remove apolipoprotein B (apoB)-containing particles (i.e., LDL and VLDL) [35]. Total cholesterol levels in the supernatants were measured by an enzymatic method using a commercially available kit (Wako Pure Chemical Co., Osaka, Japan). ApoA-I levels were quantified enzymatically in HDL-containing preparations on a Cobas Fara analyzer (Roche Diagnostics Systems, Inc) using reagents from Sigma Aldrich (St. Louis, MO).

Aliquots of total serum or the LDL- and VLDL-depleted, HDL-containing fractions were subjected to LC-MS-MS analysis on a Thermo Finnigan TSQ 7000 triple quadrupole mass spectrometer, operating in a Multiple Reaction Monitoring (MRM) positive ionization mode, using a modified version of the protocol described by Bielawski et al. [36]. Briefly, CCHS samples (50 μl diluted 1:2 with Dulbecco's phosphate-buffered saline [DPBS]) were fortified with the internal standards (ISs: C17 base D-erythro-sphingosine (17CSph), C17 S1P (17CS1P), N-palmitoyl-D-erythro-C13 sphingosine (13C16-Cer) and heptadecanoyl-D-erythro-sphingosine (C17-Cer)), and extracted with ethyl acetate/iso-propanol/water (60/30/10 v/v) solvent system. After evaporation and reconstitution in 100 μl of methanol, samples were injected on the HP1100/TSQ 7000 LC/MS system and gradient eluted from the BDS Hypersil C8, 150 × 3.2 mm, 3 μm particle size column, with 1.0 mM methanolic ammonium formate/2 mM aqueous ammonium formate mobile phase system. Peaks corresponding to the target analytes and internal standards were collected and processed using the Xcalibur software system. Quantitative analysis was based on calibration curves generated by spiking an artificial matrix with known amounts of the target analyte synthetic standards and an equal amount of the internal standards (ISs). The target analyte/IS peak areas ratios were plotted against analyte concentration. The target analyte/IS peak area ratios from the samples were similarly normalized to their respective ISs and compared to the calibration curves, using a linear regression model.

### Statistical analysis of data

A one-way ANOVA model was fit to each response in order to look for differences in group means. The Studentized residuals were calculated and assessed for the model assumptions of normality and constant variance. Following a significant result, Tukey's adjustment for multiple comparisons was used to control the Type I error rate associated with the corresponding pairwise comparisons. Hypothesis tests were conducted at level of significance 0.05.

### S1P- and DH-S1P-fortification of HDL

HDL (1.063-1.21 g/ml density fraction) was purified from human plasma by sodium bromide density-gradient centrifugation as described in Wasan et al. [37] and dialyzed against DPBS, 0.03 mM EDTA. Following dialysis, total cholesterol, LDL-C and HDL-C were determined using a lipid profile chip (Cholestech, Hayward, CA). No LDL-C was detected in the purified HDL fraction. Protein content of HDL was measured by Bio-Rad DC assay (Bio-Rad, Hercules, CA). Sphingolipid content of HDL was measured by LC-MS-MS by the MUSC Lipidomics Core Facility according to previously reported methods [5]. S1P- and DHS1P-fortified HDL were prepared by preincubation of native HDL overnight at 4°C with varying amounts of S1P or DH-S1P (0-1.38 μg S1P/mg HDL protein) followed by dialysis against DPBS, 0.03 mM EDTA to remove free sphingolipds. Sphingolipid content of fortified HDL was measured by LC-MS-MS.

### Transendothelial electrical resistance (TEER) assay

TEER was measured by electrical cell substrate impedance sensing (ECIS) [38] as described previously [5]. Briefly, human umbilical vein endothelial cells (HUVEC) (Cascade Biologics, Inc., Portland, OR) maintained in EGM-2 medium were seeded into wells of ECIS 8W10E+ electrode arrays at a density of 1 × 10^5 ^cells per well. Arrays were pre-coated with human plasma fibronectin (Invitrogen, Carlsbad, CA) at 100 μg/ml in 0.15 M NaCl, 0.01 M Tris, pH 8.0. Cells were cultured in EGM-2 medium and impedance measured every five min at 15 kHz frequency. When the electrical resistance reached a maximal plateau (~3 days) the medium was replaced with serum-free endothelial basal medium (EBM; Lonza) containing 1X penicillin-streptomycin-glutamine (Invitrogen). Electrical resistance was monitored until a minimal plateau was reached (~24 h). Effectors (i.e., S1P fortified-HDL, dihydro-S1P (DH-S1P)-fortified HDL, S1P-albumin (fatty acid free bovine serum albumin from Sigma), DH-S1P-albumin or albumin in DPBS) were introduced into the culture medium by removing a volume corresponding to that of the effector to be added. S1P (i.e., D-*erythro*-sphingosine-1-phosphate) and DH-S1P (i.e., dihydro-D-*erythro*-sphingosine-1-phosphate) were purchased from Avanti Polar Lipids, Inc. (Alabaster, AL). The maximum volume of each effector added did not exceed 1/25 of the 400 μl volume of conditioned culture medium in each well. For experiments evaluating the effects of the S1P1 antagonist W146 (Avanti Polar Lipids) and the S1P1/S1P3 antagonist VPC23019 (Avanti Polar Lipids) on TEER, antagonist stocks (1 mM in 5% acidified DMSO, 4 mg/ml BSA) were diluted 1:100 into the conditioned EBM at the same time that S1P or HDL was added.

## List of abbreviations used

S1P: sphingosine 1-phosphate; DH-S1P: dihydro sphingosine 1-phosphate, HDL: high density lipoproteins; HDL-C: HDL cholesterol; apoA-I: apolipoprotein A-I; IHD: ischemic heart disease; CCHS: Copenhagen City Heart Study; LC-MS-MS: Liquid chromatography/mass spectrometry; TEER: transendothelial electrical resistance; EC: endothelial cell.

## Competing interests

The authors declare that they have no competing interests.

## Authors' contributions

KMA, WSA, AAS and ATR conceived and designed the study, interpreted data, and drafted the manuscript. AAS worked to define the study population. BGN and ATH provided serum samples for the studies from the Copenhagen City Heart Study and participated in editing of the manuscript. SDY, JLB and KSN performed statistical analysis and participated in the interpretation of data and editing of the manuscript. BAW and PJG performed HDL purification, S1P supplementation of HDL and ECIS experimentation. BAW participated in drafting and editing of the manuscript. All authors read and approved the final manuscript.

## Supplementary Material

Additional file 1 **Evaluation of the effects of C24:1 ceramide on endothelial barrier**. Confluent endothelial cell (EC) monolayers were grown under serum-free conditions until a minimal TEER plateau was reached. EC monolayers were incubated with varying concentrations of C24:1 ceramide [333-3000 nM) or S1P [111 nM]. Each of the TEER tracings shown is an average of two replicates per condition. Impedance values were normalized by dividing each value by the level of impedance measured just prior to the addition of effectors. As a control, EC monolayers were treated with delipidated albumin (Control), a concentration corresponding to the amount of BSA carrier used for the highest concentration of C24:1 ceramide tested. Click here for file
