# Supplementary figures and images for: S1P, dihydro-S1P and C24:1-ceramide levels in the HDL-containing fraction of serum inversely correlate with occurrence of ischemic heart disease
Source: Lipids Health Dis. 2011 May 9;10:70. doi: 10.1186/1476-511X-10-70 (PMC3116499; doi:10.1186/1476-511X-10-70)

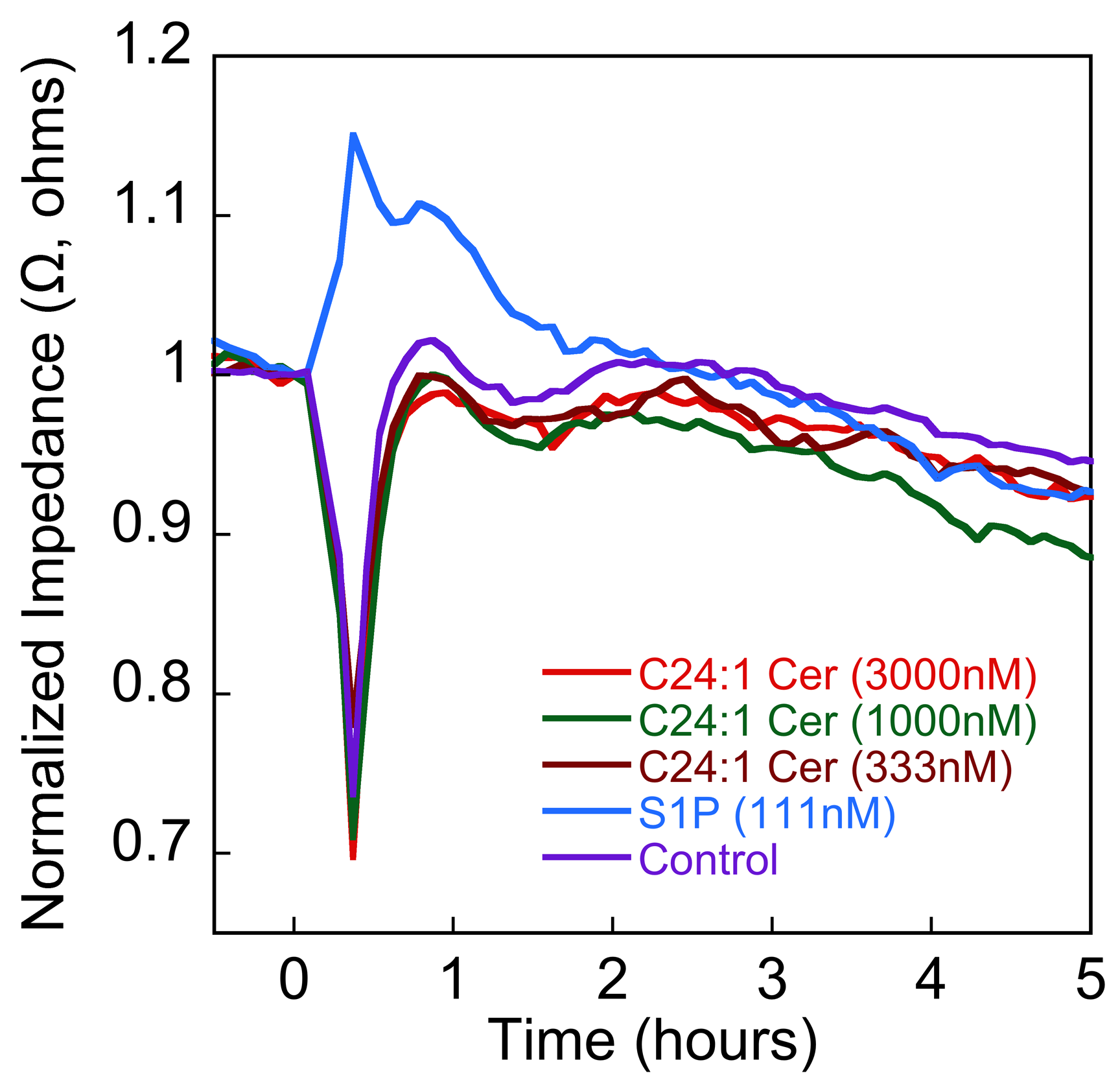

Supplement: Additional file 1 — Evaluation of the effects of C24:1 ceramide on endothelial barrier. Confluent endothelial cell (EC) monolayers were grown under serum-free conditions until a minimal TEER plateau was reached. EC monolayers were incubated with varying concentrations of C24:1 ceramide [333-3000 nM) or S1P [111 nM]. Each of the TEER tracings shown is an average of two replicates per condition. Impedance values were normalized by dividing each value by the level of impedance measured just prior to the addition of effectors. As a control, EC monolayers were treated with delipidated albumin (Control), a concentration corresponding to the amount of BSA carrier used for the highest concentration of C24:1 ceramide tested. [file 1476-511X-10-70-S1.TIFF]
